# Circulation EBV Mir-Bart-7 Relating to Clinical Manifestation in Nasopharyngeal Carcinoma

**DOI:** 10.31557/APJCP.2020.21.9.2777

**Published:** 2020-09

**Authors:** Tirta Wardana, Lisa Gunawan, Cita Herawati, Risky Oktriani, Sumadi Lukman Anwar, Indwiani Astuti, Teguh Aryandhono, Sofia Mubarika

**Affiliations:** 1 *Department of Biomedicine, School of Dentistry, Faculty of Medicine, Jenderal Soedirman University, Purwokerto, Indonesia. *; 2 *Postgraduate Student, Center for Biotechnology, Universitas Gadjah Mada, Yogyakarta, Indonesia. *; 3 *Department of Ear, Nose and Throat, Dharmais National Cancer Hospital, Jakarta, Indonesia. *; 4 *Department of Biochemistry, Faculty of Medicine, Public Health and Nursing, Universitas Gadjah Mada, Yogyakarta, Indonesia.*; 5 *Division of Surgical Oncology, Department of Surgery, Faculty of Medicine, Public Health and Nursing, Universitas Gadjah Mada, Yogyakarta, Indonesia. *; 6 *Department of Pharmacology and Therapy, Faculty of Medicine, Public Health and Nursing, Universitas Gadjah Mada, Yogyakarta, Indonesia. *; 7 *Department of Histology and Cell Biology, Faculty of Medicine, Public Health and Nursing, Universitas Gadjah Mada, Yogyakarta, Indonesia. *

**Keywords:** EBV, microRNA, BART7, nasopharyngeal carcinoma, circulation

## Abstract

**Objective:**

Nasopharyngeal Carcinoma (NPC) is an endemic head and neck malignancy in Asia Pacific regions that is associated with chronic infection by Epstein-Barr virus (EBV). EBV miR-BART-7 is a microRNA (miRNA) encoded by EBV that regulates malignant behavior of NPC. However, the role and function of miR-BART7 are not clear, particularly the relation of circulating levels and patient’s clinical presentation.

**Methods:**

Circulating miR-BART-7 levels were measured by using qRT-PCR and were correlated with clinical and pathological data. Result: Of 52 NPC patients included in this study, 85% were diagnosed in the late stages (Stage III-IV). 73% of tumors were non-keratinizing undifferentiated NPC, 92% of tumors were WHO class III histology and all cases were EBV-IgA positive. Over-expression of miR-BART7-3p was correlated with positive regional lymph nodes in newly diagnosed (4.61 fold changes, p<0.05). Conclusion: Over-expression of circulating EBV miR-BART7 correlated with positive regional lymph nodes reflecting the diagnostic and prognostic values of circulating miR-BART7 for patients with NPC.

## Introduction

Nasopharyngeal carcinoma (NPC) is a epidemic malignancy which high incidence and mortality rates in Asia including in China, Singapore, Malaysia, Vietnam, and Indonesia (Adham et al., 2012). In Indonesia, new cases and mortality rates increase every year although specific interventions both clinical management and public health awareness have been done (Mo et al., 2012)(World Health Organization, 2019). In Indonesia, 17,992 new NPC cases were diagnosed yearly with associated mortality of 11.204 (World Health Organization, 2019). The carcinogenesis of NPC is associated with multistep process of genetic and epigenetic alterations with close association with early-stage Epstein Barr Virus (EBV) infection. 

The Epstein-Barr virus (EBV) is a double-stranded DNA virus which latently infects more than 90% population worldwide (Sarwari et al., 2016). EBV is frequently associated with an increased risk of several well-defined malignancies, including Burkitt lymphoma, Hodgkin’s lymphoma, Nasopharyngeal carcinoma, gastric cancer, and distinct B-/T-NK-cell lymphoma (Barukcic, 2019). In NPC patients, the involvement of EBV infection is usually detected using serological testing to measure elevated IgA antibody responses Viral Capsid Antigen (VCA), Membrane Antigen (MA), and Nuclear Antigens (EBNA) (Middeldorp, 2015).

EBV infection uses and disrupts some intracellular and membranous cellular protein signaling pathways as well as non-coding RNAs to support its replication and latent infection. MicroRNAs (miRNAs) are small non-coding RNAs (18-24 nucleotides) that function as a regulator of post-transcriptional protein expression through inhibition and degradation of mRNA targets (Mo et al., 2012). Several host and EBV-encoded miRNAs have been associated with NPC carcinogenesis by targeting hundreds of mRNA involving in cellular pathways leading to cancer initiation and progression (Oliveto et al., 2017). miRNAs are relatively stable, not easily degraded, as well as resistant to ribonuclease, and extreme physic-chemical conditions such as heating, freezing, and pH (O’Brienet al., 2018; Hernandez-Ontiveros et al., 2013). miRNAs can be detected in the extracellular fluids such as blood circulation, urine, tears, saliva, and breast milk (Weber et al., 2010; Anwar et al., 2019). miRNAs have been shown to play as a clinical biomarker in cancer including in NPC (Cosmopoulos et al., 2009) and miR-BART7 (BamHI-A rightward transcript) is one of EBV-encoded miRNAs that was shown to circulate in the human body with potential as diagnostic or prognostic marker (Zhang et al., 2015; Ramayanti et al., 2019). Circulating EBV miRNAs may more directly reflect tumor viable activity as compared to circulating EBV DNA, which shown to derive from dying apoptotic tumor cells (Allen and Dennis, 2002, Mo et al., 2012; Kim et al., 2017) 

EBV miR-BART-7 was shown to target several host RNA species including miRNAs and mRNA (Kang et al., 2017; Wang et al., 2018). Previous studies have shown that miR-BART7 induce invasion, migration and proliferation of cancer cells (Cai et al., 2015). In this study, we measured the expression levels of circulating EBV miR-BART-7 in NPC patients in relation to pathological and clinical characteristics of the patients and compared these with healthy individuals as controls.

## Materials and Methods

*Study population*

Patients diagnosed with nasopharyngeal carcinoma were recruited to participate in this study. Sample collection procedure was approved by ethic committee Faculty Medicine Universitas Gadjah Mada, Yogyakarta, Indonesia(KE/FK/0892EC 14 Augusts 2019). In total, peripheral blood samples were collected from NPC patients at diagnosis (n=52) and healthy non-tumor controls (n=30) were from National Dharmais Cancer Hospital, Jakarta Indonesia. The diagnosis of NPC was confirmed by histopathology examination. In addition, the presence of EBV was confirmed molecular techniques using serological assay.NPC patients were older than 18 years and were able to provide informed consent prior to being recruited. Calculation of sample size using effect size 0.5 of Cohen’s approach with confidence level 95%, α=0.05. The statistical power 0.9 determined that a sample size was needed of ≥ 48 patients. Clinicopathological variables include staging, lymph nodes, tumor size, local and distant metastasis, pathological characteristics of NPC participants in this study are summarized in Table 1. 

*miRNA Extraction and synthesis cDNA*

Total of 200 µl plasma was extracted using miRCURY-Biofluids, Exiqon Denmark (Cat no. 30112). cDNA synthesis using universal cDNA synthesis kit II (Cat No. 203301, Exiqon) was done on a Biorad C1000 thermal cycler with a condition by 42°C for 60 minutes, 95°C for 5 minutes and 4°C. All conditions and procedures were according to the recommendation by the manufacture. 

*Quantitative PCR (qPCR)*

Quantification process of miRNA using Exilent SYBR Green master mix, 2,5 mL (Cat No. 203402, Exiqon), primer LNA EBV miR-BART-7-3p (Cat no. YP00205738, Exiqon), Sp6 (Cat no. YP00203954, Exiqon) and reference gene miR-16 (cat no YP00205702, Exiqon). cDNA was diluted 1:80 from 5 µl cDNA by adding nuclease-free water.A volume of 4 µl of dilution was used for the quantification process using real-time PCR Biorad CFX 96 with a condition: 95°C for 10 minutes, 95°C for 10 seconds, 40 cycles amplification 58°C, and 60°C, 1-minute ramp-rate 1,6°C/s. All procedures were done following the manufacture’s recommendation. 

*EBV antibody assay*

IgA (EBV-EBNA+VCAp-18+EA) Antibody assay levels were measured in plasma sample using Elisa (Greiner Labortechniek; Frickenhausen, Germany). Serology examination is carried out to find out the sero activity of IgA (EBNA-VCAp-18 + EA) and standard inspection procedures in NPC cases. IgA antibody levels against the EBV-EBNA, VCAp-18 and EA was measured according previously protocols(Fachiroh et al., 2008). The cut-off from the calibrator is checked twice. The cut of value for each plate is defined as calculating the mean absorbance of OD450.

*Data Analysis*

Data analysis of EBV miR-BART7 expression used relative quantification Livak’s methods to know alterations expression in NPC (Livak and Schmittgen, 2001). Data analysis fold change of expression used GenEx 7, MultiD. Fold change and statistical analysis was conducted to define the correlation between BART7 expression and clinical status using the student t-test. Correlation of expression between miR-BART7 and EBV protein used Spearman rank correlation. All statistical test with 2-sided and a p-value <0.05 was indicated as statistical significance. Statistical analysis was performed using GraphPad Prism 6 (La Jolla CA, USA).

## Results

*Study population*

Participants’ demographical and clinical characteristic was summarized in Table 1. In total, 82 peripheral blood samples were collected consisting of 30 healthy controls (HC) and 52 nasopharyngeal carcinoma cases (NPC). The percentage of male diagnosed more than female was 67%. NPC patients were grouped based on histology and pathology anatomy according to the World Health Organization (WHO) standard. According to histological characteristics, >90% of tumors were WHO type III. EBV related IgA antibodies were measured in patients with NPC (EA, EBNA and VCA immunoassay). More than 95% of patients showed positive IgA-EBV serology status (Table 1). 

*EBV antibody assay*

Validation of EBV-IgA serology in 52 plasma NPC patients was done to determine their relationship to the presence of NPC. IgA serological analysis of EA, EBNA, and VCA has been measured using Elisa reader. EBV IgA antibodies were detected in plasma of participants with EBV-EA in 43 (83%) of 52 patients, EBV-EBNA in 36 patients (69%) and EBV-VCA in 47 patients (90%). All participants confirmed positive IgA serology for one or more EBV marker.

*Quality control plasma-miRNA expression analysis*

Expression analysis of EBV miR-BART7 was conducted using qRT-PCR with analysis methods relative quantification using delta cycle quantification (Cq) (miR-BART7 – miR-16-5p). miR-16-5p was used as a reference gene for profile expression quantification. The CQ of 52 sample plasma NPC and 30 HC included in the study miR-BART7 34.97 (±2.262) and 36.016 (±3.93). Unisp6 RNA Spike-in synthesis in a template of each plasma sample was used as quality control from miRNA extraction step, synthesis cDNA and qPCR. Target quantification used LNA primer showing in graft Melt Peak with at 74°C as indicate the specific target.

*Circulating level expression miR-BART7 across all clinical data*

**Figure 1:**
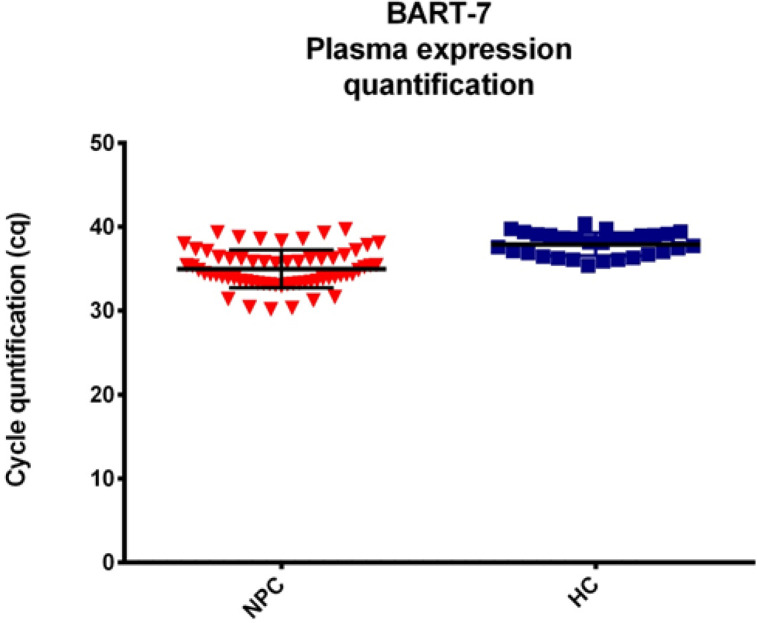
Quantification of Circulation miR-BART-7, It was Indicated Resulting from Cycle Quantification (CQ) from Two Type Sample Nasopharyngeal Carcinoma (NPC) and Healthy Control (HC)

qRT-PCR quantification showed results in the form of a cycle quantification (CQ), miR-BART7 can be detected on the circulation of NPC patients. The results of the CQ can be seen in Figure 1, it’s indicated abundance expression of BART7 on NPC with mean ±SD 34.97 (±2.262) and HC 36.016 (±3.93). Reference gene in circulation NPC used miR-16 with mean ±SD 23.96 (±0.959) and HC 23.19 (±0.063). Reference gene miR-16 is stable can be seen from the range of differences in expression in NPC and HC sample close to 0. 

Relative expression analysis was conducted based on clinical data using software GenEx 6 MultiD. Statistical analysis used T-Test to compare differential group. Table 2 shows the results of the differential expression of miR-BART7 based on clinical data of participants. There are 4 groups with the clinical status that are used as a basis for grouping namely Health and NPC, N-Regional Lymph Nodes, T-Primary Tumor and Stage. 

**Figure 2:**
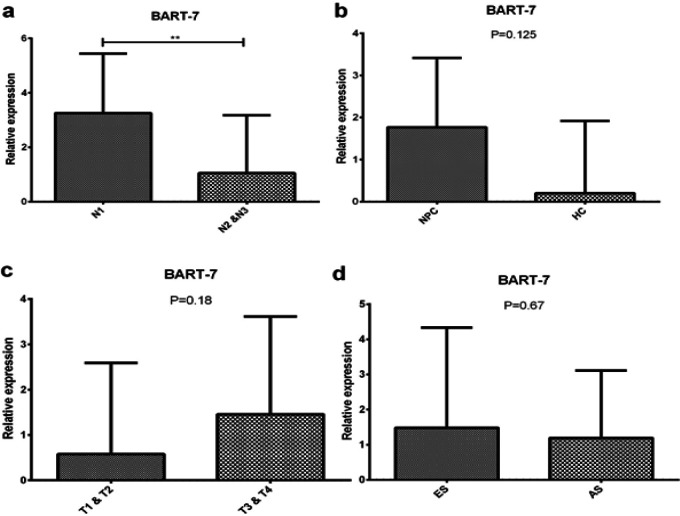
Relative Expression BART7 on Plasma Nasopharyngeal Carcinoma Based on Clinical Status, a. N-regional lymph nodes: over-expression miR-BART7 on N1, b. over-expression miR-BART7 on plasma NPC, c. T-Primary tumor: BART-7 over-expression on circulating T1 and T2 status and d. Stage: over-expression BART7 in early-stage compared with the advanced stage

Statistical analysis showed that there were no significant differences in the expression on several variables such as cases, T-Primary tumor and stage. Whereas, There is a significant expression on miR-BART7 with a difference in N-Regional Lymph Nodes status. miR-BART7 with N1 and N2 showed over-expression with fold change 4.61 (P<0.05). Alterations in expression of miR-BART7 in circulation occurred on each group NPC. In circulation, BART7 expression increased with fold change 2.15 (P=0.125), T-Primary Tumor status increased by 1.84 (p-value =0.18) in T1 and T2. Furthermore, circulation expression of BART7 increased on early-stage by fold change 1.22 (p-value = 0.67). The graph of expression analysis can be seen in figure 2. There are no significant relationships between EBV miR-BART7 with any pair of variables in the correlation of EBV protein EBNA, EA and VCA (P > 0.050).

**Table 1:** Demographical and Clinical Characteristic of the Study Population

Characteristic	N	Per cent (%)
Healthy control (HC)	30	100
Nasopharyngeal Carcinoma (NPC)	52	100
Median Age (Years), Range	47 (14-68)	
Sex		
Male	35	67%
Female	17	33%
Histology (WHO)		
WHO I	1	2%
WHO II	3	6%
WHO III	48	92%
Stage at diagnosis		
I	0	0
II	8	15%
III	16	31%
IV	28	54%
Pathology Anatomy		
Undifferentiated	10	19%
Non-Keratin, Undiff-Sub Type	38	73%
Non-Keratin, Differentiated	3	6%
Keratin	1	2%
EBV - EA		
Positive	43	83%
Negative	9	17%
EBV - EBNA		
Positive	36	69%
Negative	16	31%
EBV - VCA		
Positive	47	90%
Negative	5	10%

**Table 2:** Relative Expression of BART-7 Coding miRNA EBV in Plasma Nasopharyngeal Carcinoma

Variable	Cycle quantification microRNAs (Mean ± SD)		Fold Change	P-Value
	BART-7	miR-16		
Cases				
NPC	34.97 (±2.262)	23.96 (±0.959)	2.15	0.125
Healthy Control	36.016 (±3.93)	23.19 (±0.063)		
N- Regional Lymph Nodes				
N1 and N2	33.45 (±2.084)	24.29 (±1.615)	4.61	<0.05*
N3 and N4	35.84 (±2.852)	24.06 (±0.924)		
T- Primary Tumor				
T1 and T2	35.65 (±2.476)	23.65 (±0.992)	1.84	0.18
T3 and T4	35.12 (±3.103)	24.26 (±0.998)		
Stage				
Early Stage	35.17 (±3.522)	24.12 (±1.206)	1.22	0.67
Late Stage	35.38 (±2.588)	24.08 (±0.967)		

*differences significance P-Value

## Discussion

NPC is an epithelial malignancy of the cube-shaped nasopharyngeal region located behind the nose with a size of 4-5 cm (Pollard et al., 2016). Hidden anatomic location and symptoms are not specific makes NPC difficult to diagnose at an early stage. The International Union against Cancer (UICC) and American Joint Committee on Cancer (AJCC) have developed the TNM staging system. it becomes one of the indicators for the assessment of disease progression, prediction of prognosis, staging and selection of appropriate drugs (Pontius et al., 2017; Brierley et al., 2017). However, clinical-pathology parameters are continuously evaluated to find sensitive biomarker indicators that can improve the outcome of NPC therapy. Interestingly, EBV DNA to this day is considered as one of the right markers for diagnosis and NPC screening.

NPC secretes genetic material not only DNA and Protein but also miRNA which can interfere with host genetic regulation. Deregulation of miRNA expression is responsible for cancer growth. Increased or decreased miRNA expression plays a role in almost all cellular mechanisms including tumor growth, invasion, metastasis, proliferation and angiogenesis. EBV is the first virus known to express miRNA in the incidence of cancer (Lin and Flemington, 2011). The previous study, miRNA-EBV is represented by the sequencing method of NPC tissue (Lung et al., 2018). MiR-BART-7-encoded EBV is expressed in NPC events and has the potential to be used as a marker and therapeutic target through manipulation of miRNA expression levels in NPC. 

At the NPC incident, alterations of miR-BART-7 expression as one of the latent genes of EBV expressed. The gene encoded by EBV contributes to the formation of cancer. However, this is not yet known with certainty the clinical manifestations produced. This study is focused on understanding how changes in BART-7 expression correlated with the clinical outcome of patients. Circulation of BART-7 expression on NPC malfunction can provide an overview of the mechanism and its role. In this study, we show that there are differences in circulatory expression in BART7 and the TNM status of patients. 

At the NPC Occurrence, there were 29 miRNA-EBVs that had an increased expression of BART-7 tissue NPC (Wong et al., 2012). Increased BART-7 expression will change somatic genes by manipulating the host cell biogenesis engine. This is caused by the control process in the post transcription level. However, the relationship between EBV infection and NPC remains to be studied more comprehensive. Epithelial infection may not be a primary process in the carcinogenesis associated with viruses, because of tonsils from patients with infectious mononucleosis (IM) and normal nasopharyngeal biopsies from high-risk individuals suffering from NPC related with the existence of EBV (Young and Rickinson, 2004). Previous studies stated that increased BART-7 expression had implications for the poor prognosis of early and late-stage NPC patients (Tan et al., 2019). BART-7 is known to target WNT signalling pathways, TGF-B, and ECM (Zhang et al., 2015; Wan et al., 2015). Additionally, Over-expression of BART-7 showed its relationship to the status nodule (p <0.05) and tumor grade. NPC cells affect cell proliferation, invasion and cell migration. Moreover, changes in abnormal expression in vitro cause more resistance to cisplatin (Chan et al., 2012). BART-7 is a potential candidate marker for clinical outcome and prognosis status of NPC. 

We recognize that there are several potential weaknesses in our research. First, most of the samples recruited were advanced stages. Secondly, samples from normal subjects cannot be identified related to health conditions when collecting samples because they will greatly affect the condition of EBV infections.
